# Antifibrotic Effect of Lactulose on a Methotrexate-Induced Liver Injury Model

**DOI:** 10.1155/2017/7942531

**Published:** 2017-08-17

**Authors:** Banu Taskin, Mümin Alper Erdoğan, Gürkan Yiğittürk, Damla Günenç, Oytun Erbaş

**Affiliations:** ^1^Faculty of Medicine, Department of Dermatology, Istanbul Bilim University, Istanbul, Turkey; ^2^Faculty of Medicine, Department of Physiology, Ege University, Izmir, Turkey; ^3^Faculty of Medicine, Department of Histology and Embryology, Ege University, Izmir, Turkey; ^4^Faculty of Medicine, Department of Internal Medicine, Ege University, Izmir, Turkey; ^5^Faculty of Medicine, Department of Physiology, Istanbul Bilim University, Istanbul, Turkey

## Abstract

The most severe side effect of prolonged MTX treatment is hepatotoxicity. The aim of this study is to investigate the effect of lactulose treatment on MTX-induced hepatotoxicity in a rat model. Twenty-four male rats were included in the study. Sixteen rats were given a single dose of 20 mg/kg MTX to induce liver injury. Eight rats were given no drugs. 16 MTX-given rats were divided into two equal groups. Group 1 subjects were given lactulose 5 g/kg/day, and group 2 subjects were given saline 1 ml/kg/day for 10 days. The rats were then sacrificed to harvest blood and liver tissue samples in order to determine blood and tissue MDA, serum ALT, plasma TNF-*α*, TGF-*β*, and PTX3 levels. Histological specimens were examined via light microscopy. Exposure to MTX caused structural and functional hepatotoxicity, as evidenced by relatively worse histopathological scores and increased biochemical marker levels. Lactulose treatment significantly reduced the liver enzyme ALT, plasma TNF-*α*, TGF-*β*, PTX3, and MDA levels and also decreased histological changes in the liver tissue with MTX-induced hepatotoxicity in the rat model. We suggest that lactulose has anti-inflammatory and antifibrotic effects on an MTX-induced liver injury model. These effects can be due to the impact of intestinal microbiome.

## 1. Introduction

Methotrexate (MTX), a folic acid analogue, is an anti-inflammatory, antiproliferative, and immunosuppressive agent. MTX treatment is effective for malignancies and some chronic inflammatory diseases such as psoriasis and rheumatoid arthritis [1, 2]. Even though new biological treatments are widely used as an alternative to MTX for various dermatological diseases, MTX is still the primary choice as the cost effective and well-experienced treatment option in most cases. However, the most serious side effect of MTX is hepatotoxicity and high doses may cause steatosis, stellate cell hypertrophy, anisonucleosis, and hepatic fibrosis [3]. The exact mechanisms underlying MTX hepatotoxicity are unclear. Many studies demonstrated that MTX-induced hepatic injury may be due to oxidative stress [4]. Recently, some studies indicate that the intestinal flora might play a critical role in liver fibrosis and hepatocellular carcinoma [5–7].

As a synthetic disaccharide, lactulose has a wide range of applications in the food industry, especially as a probiotic food, and it has been used as a drug mainly for the treatment of hepatic encephalopathy and constipation [8, 9]. Although dysregulated intestinal microbiome has been found associated with complications of end-stage liver disease, there is no study about the effect of lactulose on MTX-induced liver toxicity, to the best of our knowledge. The presented study aimed to investigate the antifibrotic effects of lactulose on a methotrexate-induced liver injury rat model.

## 2. Materials and Methods

### 2.1. Animals

In this study, 24 male Sprague Dawley albino mature rats, weighing 200–220 g, were used. Animals were fed ad libitum and housed in pairs in steel cages having a temperature-controlled environment (22 ± 2°C) with 12 h light/dark cycles. The experimental procedures employed in the present study were approved by the Animal Ethics Committee. All experiments were carried out according to the Guide for the Care and Use of Laboratory Animals, as confirmed by the National Institutes of Health (US).

### 2.2. Experimental Protocol

Twenty-four male rats were included in the study. Sixteen rats were given a single dose of 20 mg/kg MTX to induce liver injury. Eight rats were given no drugs (normal group). 16 MTX-given rats were divided into two groups. Group 1 rats received lactulose 5 g/kg/day (Duphalac syrup, Abbott), and group 2 rats received saline (% 0.9 NaCl) 1 ml/kg/day. Saline and lactulose were given via oral gavage for 10 days. The animals were euthanized, and blood samples were collected by cardiac puncture for biochemical analysis. Liver was removed for histopathological and biochemical examination.

### 2.3. Histopathological Evaluation

Formalin-fixed liver sections (4 *μ*m) were stained with hematoxylin and eosine. All sections were photographed with Olympus C-5050 digital camera mounted on Olympus BX51 microscope.

Liver histopathological scoring analysis was performed according to Lobenhofer et al. The assessment was expressed as the sum of the individual score grades from 1 (minimal), 2 (mild), and 3 (moderate) to 4 (marked) for each of the following parameters from liver sections: hepatocyte necrosis, fibrosis, and cellular infiltration [10].

### 2.4. Measurement of Plasma TNF-*α* Levels

Plasma TNF-*α* levels were measured using a commercially available enzyme-linked immunosorbent assay (ELISA) kit (Biosciences). The plasma samples were diluted 1 : 2, and TNF-*α* was determined in duplicate according to the manufacturer's guide. The detection range for TNF-*α* assay was <2 pg/ml.

### 2.5. Determination of Plasma ALT Levels

Plasma ALT levels were measured using a commercially available (ELISA) kit (Uscn Life Science Inc.).

### 2.6. Determination of Lipid Peroxidation

Lipid peroxidation was determined in tissue and plasma samples by measuring malondialdehyde (MDA) levels as thiobarbituric acid reactive substances (TBARS) [11]. Briefly, trichloroacetic acid and TBARS reagent were added to the tissue samples then mixed and incubated at 100°C for 60 min. After cooling on ice, the samples were centrifuged at 3000 rpm for 20 min, and the absorbance of the supernatant was read at 535 nm. MDA levels of tissue were calculated from the standard calibration curve using tetraethoxypropane and expressed as nmol/g protein.

### 2.7. Evaluation of Plasma Pentraxin 3 Levels

Plasma pentraxin 3 (PTX3) levels were measured in each 100 *μ*l sample by a standard ELISA apparatus at 450 nm by using a PTX3 kit (Uscn Life Science Inc., Wuhan, China). PTX3 levels were determined in duplicate according to the manufacturer's guide.

### 2.8. Statistical Analysis

Data analyses were performed using SPSS version 15.0 for Windows. The groups of parametric variables were compared by Student's *t*-test and analysis of variance (ANOVA). The groups of nonparametric variables were compared by Mann–Whitney *U* test. Results were given as mean ± standard error of mean (SEM). A value of *p* < 0.05 was accepted as statistically significant. *p* < 0.001 was accepted as statistically highly significant.

## 3. Results

### 3.1. Histological Analysis

The results of histological injury scores of the groups are summarized in Table 1.

**Figure 1:**
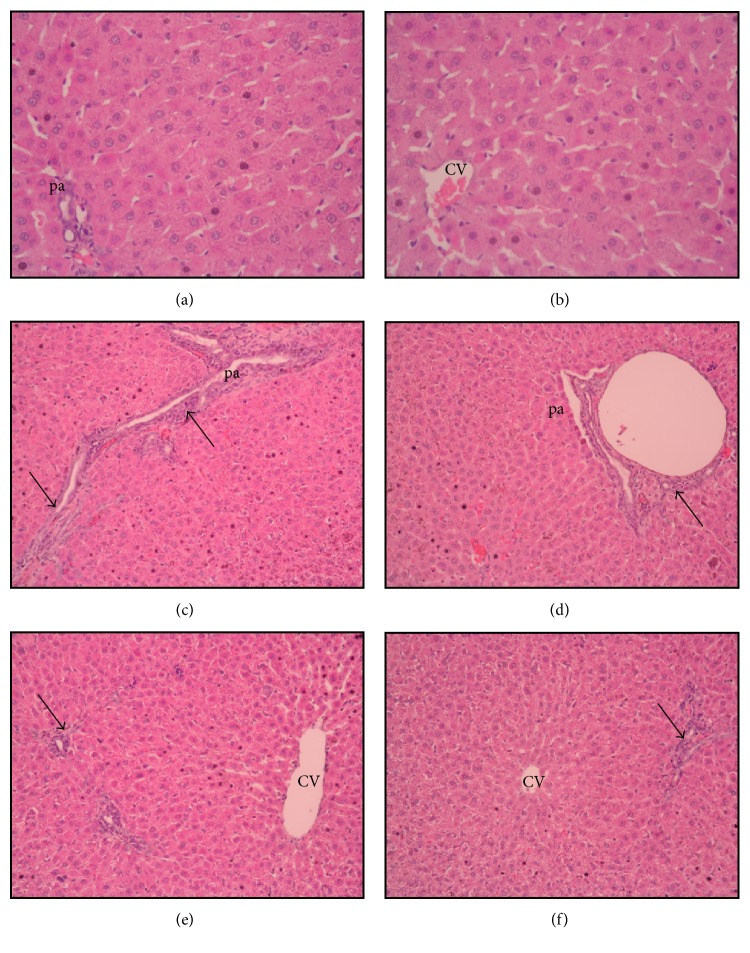
Liver histopathology H&E (×20 magnification), (a–b): normal liver, (c–d): bridging necrosis, fibrosis, and cellular infiltration in portal area (pa) (arrow), central venous (CV), (e–f): decreased bridging necrosis, fibrosis, and cellular infiltration in portal area (pa) (arrow).

The liver sections of the normal group have a normal histological appearance (Figures 1(a) and 1(b)). The histological appearance of the liver sections from the MTX + lactulose group (Figures 1(e) and 1(f)) is significantly better than that of the liver sections from the MTX + saline group (Figures 1(c) and 1(d)).

### 3.2. Biochemical Analysis

As shown in Table 2, MTX + saline group showed a significantly higher levels of ALT, plasma TGF-*β*, plasma MDA, plasma TNF-*α*, and liver MDA activity compared to the MTX + lactulose group.

## 4. Discussion

The most severe side effect of prolonged MTX treatment is hepatotoxicity. Reducing this side effect significantly improves patient well-being and the treatment success [12]. This is the first study in literature investigating the effect of lactulose treatment on MTX-induced hepatotoxicity in a rat model.

In the study, MTX-treated group demonstrated various liver histological changes such as hepatocyte necrosis, fibrosis, and an increased cellular infiltration. These results comply with previous studies [12, 13]. Moreover, we correlated these histological changes with serum and biochemical studies that also suggested liver injury.

Our observations clearly demonstrated that MTX treatment increases serum levels of ALT, as shown by previous studies [13–15]. We also found that systemic inflammatory response indicator TNF-*α* has increased due to MTX administration and has been decreased by lactulose treatment.

In this study, we postulate that MTX administration triggered serum TGF-*β* increase, indicating the role of this cytokine in MTX-induced hepatotoxicity. TGF-*β* is a central regulator in chronic liver disease and contributes to all stages of disease progression from initial liver injury to fibrosis and carcinoma [16], while lactulose depresses the TGF-*β* response.

Another important finding of the study is that MTX exposure triggered increase in plasma PTX3, which is a glycoprotein in the PTX family and plays an important role in the primary inflammatory response [17]. Recently, PTX3 has been used as a biomarker of liver diseases [18, 19]. We suggest that PTX3 can be used as a marker for MTX-induced hepatotoxicity and lactulose treatment can reduce PTX3 plasma levels.

We observed that MTX administration significantly increased both plasma and tissue MDA in comparison to the normal group. Hadi et al. reported that MTX treatment of rats led to increased serum and tissue MDA levels [20] and there are many studies showing that antioxidant agents can reduce high levels of MDA [21]. Here, we demonstrated that administration of lactulose can also reduce oxidant parameters (tissue and plasma MDA levels).

The mechanism of MTX hepatotoxicity is still not fully understood and may include intracellular accumulation of MTX polyglutamate and associated folate depletion, generation of oxidative stress, and activation of proinflammatory cytokines as well as genetic polymorphism [12, 18]. Furthermore, many studies indicate that the intestinal flora might play a critical role in liver fibrosis and hepatocellular carcinoma [5–7]. Yu et al. reported that consumption of host microflora suppresses tumor formation in hepatocarcinogenesis with a toxic rat model. They suggest that improvement of the gastrointestinal system blood flow, reducing gut damage, and lessening the gut translocation of endotoxin may improve liver function in patients with cirrhosis with potential to progress into HCC [22]. In another study, Zhang et al. demonstrated that dysbiosis induction is sufficient to induce hepatocarcinogenesis with increased portal LPS levels. The finding was supported by the dramatic relief of enteric dysbacteriosis, hepatic inflammation, and the diminished growth of the liver tumor with probiotic treatment [23]. Lactulose has been used as a drug mainly for the treatment of constipation and hepatic encephalopathy [8, 9]. It has been suggested that lactulose may prevent endotoxin absorption in the intestine. Lactulose reduces both blood endotoxin levels and liver damage [24]. In a rat model, lactulose administration increases liver regeneration after hepatectomy by inducing hydrogen as a result of decreasing the oxidative stress response and excessive inflammatory response [22]. In our study, as all the histological and biochemical results clearly demonstrated, we evaluate that lactulose treatment has antifibrotic effects on MTX-induced hepatotoxicity in rats and this effect of lactulose can be due to impact on intestinal microbiome.

## 5. Conclusion

Hepatotoxicity is a dose-limiting side effect in long-term MTX treatments used in dermatological or other inflammatory diseases. In this study, we observed a beneficial effect of lactulose with MTX hepatotoxicity and we think that our findings can illuminate new studies about its use to reduce the mentioned hepatotoxicity.

## Figures and Tables

**Table 1:** 

Hepatocyte necrosis	0.25 ± 0.2	2.3 ± 0.25^∗∗^	0.8 ± 0.3^#^
Fibrosis	0.38 ± 0.18	2.1 ± 0.3^∗^	0.8 ± 0.1^#^
Cellular infiltration	0.2 ± 0.1	1.38 ± 0.16^∗^	0.9 ± 0.35^#^

^∗∗^ *p* < 0.0001, MTX + lactulose group compared with normal group. ^∗^*p* < 0.01, MTX + lactulose group compared with normal group. ^#^*p* < 0.05, MTX + lactulose group compared with MTX + saline group.

**Table 2:** 

	Normal	MTX + saline	MTX + lactulose
Plasma TGF-beta (pg/ml)	5.8 ± 0.9	48.5 ± 4.2^∗∗^	26.1 ± 4.4^#^
Plasma MDA (nM)	41.09 ± 5.1	216.8 ± 12.6^∗∗^	105.9 ± 10.07^##^
Plasma TNF-alpha (pg/ml)	21.9 ± 3.1	88.5 ± 7.2^∗∗^	35.03 ± 6.4^#^
Plasma pentraxin 3 (ng/ml)	1.17 ± 0.12	3.1 ± 0.34^∗∗^	1.5 ± 0.71^##^
ALT (IU/l)	22.5 ± 3.08	74.6 ± 4.4^∗∗^	48.9 ± 7.01^#^
Liver MDA (nmol/g tissue)	22.4 ± 2.5	71.8 ± 6.18^∗∗^	55.21 ± 9.2^#^

^∗∗^ *p* < 0.0001, MTX + lactulose group compared with normal group. ^##^*p* < 0.0001, MTX + lactulose group compared with MTX + saline group. ^#^*p* < 0.05, MTX + lactulose group compared with MTX + saline group.
